# Does radiofrequency radiation impact sleep? A double-blind, randomised, placebo-controlled, crossover pilot study

**DOI:** 10.3389/fpubh.2024.1481537

**Published:** 2024-10-29

**Authors:** Nicole Bijlsma, Russell Conduit, Gerard Kennedy, Marc Cohen

**Affiliations:** ^1^School of Health and Biomedical Sciences, RMIT University, Bundoora, VIC, Australia; ^2^Australian College of Environmental Studies, Warrandyte, VIC, Australia; ^3^School of Science, Psychology and Sport, Federation University, Mount Helen, VIC, Australia; ^4^Austin Health, Institute for Breathing and Sleep, Heidelberg, VIC, Australia; ^5^The Extreme Wellness Institute, Melbourne, VIC, Australia

**Keywords:** electropollution, electromagnetic fields, heart rate variability, insomnia, non-ionising radiation, sleep, sleep EEG, Wi-Fi

## Abstract

The most common source of Radiofrequency Electromagnetic Field (RF-EMF) exposures during sleep includes digital devices, yet there are no studies investigating the impact of multi-night exposure to electromagnetic fields emitted from a baby monitor on sleep under real-world conditions in healthy adults. Given the rise in the number of people reporting to be sensitive to manmade electromagnetic fields, the ubiquitous use of Wi-Fi enabled digital devices and the lack of real-world data, we investigated the effect of 2.45 GHz radiofrequency exposure during sleep on subjective sleep quality, and objective sleep measures, heart rate variability and actigraphy in healthy adults. This pilot study was a 4-week randomised, double-blind, crossover trial of 12 healthy adults. After a one-week run-in period, participants were randomised to exposure from either an active or inactive (sham) baby monitor for 7 nights and then crossed over to the alternate intervention after a one-week washout period. Subjective and objective assessments of sleep included the Pittsburgh Insomnia Rating Scale (PIRS-20), electroencephalography (EEG), actigraphy and heart rate variability (HRV) derived from electrocardiogram. Sleep quality was reduced significantly (*p* < 0.05) and clinically meaningful during RF-EMF exposure compared to sham-exposure as indicated by the PIRS-20 scores. Furthermore, at higher frequencies (gamma, beta and theta bands), EEG power density significantly increased during the Non-Rapid Eye Movement sleep (*p* < 0.05). No statistically significant differences in HRV or actigraphy were detected. Our findings suggest that exposure to a 2.45 GHz radiofrequency device (baby monitor) may impact sleep in some people under real-world conditions however further large-scale real-world investigations with specified dosimetry are required to confirm these findings.

## Introduction

Sleep is an important biological function and critical to maintain homeostasis and sleep disturbances are a major risk factor for cardiovascular disease, metabolic disorders and mortality (1). Chronic sleep disturbances adversely affect neurological functioning such as memory formation (2), sustained attention (3) and other higher cognitive functions (4), as well as strongly associated with the development of Alzheimer’s disease (5). In children and young adults, disturbed sleep is reportedly associated with mental health disorders (6), depression (7) and impaired academic performance (8). Over the last two decades, the prevalence rate of sleep disorders has significantly increased currently affecting four out of every 10 Australians with considerable impact on social, financial and health-related costs (9).

The rise in sleep disturbances coincides with the deployment of billions of mobile phones worldwide (10). Despite proliferation of these wireless communication devices and networks resulting in an increased exposure to radiofrequencies by 18 orders of magnitude (11), the relationship between RF-EMF exposure and sleep remains unclear. Sleep problems are the most commonly reported complaints attributed to RF-EMF exposure (12–14) and multiple surveys suggest that RF-EMF exposure is closely linked to symptom reporting (15–17). While sleep disturbances are highly prevalent in young adults (18) who coincidentally also spend the highest screen time accessing digital devices (19), epidemiological surveys prone to respondent bias, rarely use clinically relevant outcome measures. Furthermore, experimental research on RF-EMF exposure and sleep is complex and far from conclusive (20). Most experimental studies exploring the impact of pulse-modulated radiofrequencies on sleep quality are conducted in a highly controlled research environment using near-head exposure to mobile phones. Such studies reveal inconsistent associations, lack generalisability, with limited sample size and short-term duration or no follow-ups (21–28). Furthermore, it has been well established that sleep in a sleep laboratory is distorted, especially over a single night (29). It is also suggested that studies focus on real world settings rather than simulated electromagnetic fields as real-life signals are highly variable with unpredictable changes in intensity and waveforms which renders them more biologically active (30). To date, no study has examined the impact of repeated exposure to 2.45 GHz radiation on sleep in real-world situations, despite this type of radiation becoming ubiquitous in modern households.

The uncertainty around the impact of RF-EMFs on sleep is compounded by the uncertainties surrounding the mechanisms of action. According to a recent systematic review, exposure to pulsating RF-EMFs in selective bands increased the EEG power during sleep, however their effect on sleep architecture or clinical sleep outcomes remains unclear (20). It has been suggested that RF-EMFs may impact sleep through multiple mechanisms including direct exposure to pulse-modulated RF-EMFs influencing EEG architecture (31–33), induced melatonin suppression from exposure to blue light at bedtime (34), device-induced arousal reducing the ability to fall asleep, or other factors related to the use of mobile phones such as media use before bedtime or after lights out (27). The proximity and timing of exposure may also be important with a large systematic review and meta-analysis involving 125,198 children concluding that sleep disturbances and daytime sleepiness were significantly more common when a device was in the bedroom, even when the child did not use the device at night (35). Further evidence suggests that sleep outcomes are more likely to be adversely affected by RF-EMF when exposures occur throughout the night (21). Yet, physiological studies on the effects of Wi-Fi related frequencies on sleep are generally carried out under laboratory conditions rather than real-world settings and report considerable variation on the relationship between RF-EMF and sleep architecture (36).

We aimed to address the gaps in current knowledge using a robust, double-blind, randomised, placebo-controlled, crossover methodology in a real-world setting, to explore the effects of exposure from a commonly used radiofrequency device used over multiple nights on clinically relevant sleep outcomes in healthy adults. This is a novel approach as the experimental protocol involved participants’ own homes and natural sleeping environments with a readily available consumer electronic device, hence obtaining ecologically-valid, empirical evidence.

## Materials and methods

### Study design

#### Radiofrequency device, exposure set-up, and power dosimetry

The study involved a randomised, double-blind, placebo-controlled, crossover design over 4 weeks on healthy adults at their homes in Melbourne, Australia. We compared 7 consecutive all-night exposure to either an active or inactive (sham) pulse-modulated radiofrequency device. The device used was a commercially available Uniden baby monitor (BW 3001 model), consisting of a digital wireless monitor and digital wireless camera with two-way walkie talkie capability. This device has a transmitting power of 15 dBm and employs a frequency range of 2.4 to 2.4835 GHz using a frequency-hopping spread spectrum system (FHSS) with Gaussian Frequency Shift Keying (GFSK) modulation to avoid interference. The units were tested prior to randomisation to determine the level of radiation emitted. This was done by placing them two metres apart and using a Gigahertz HF59B Analyser with UBB27 omnidirectional antenna (frequency range between 27 MHz to 3.3 GHz) and a Gigahertz HFW59D Analyser with UBB2410 omnidirectional antenna (frequency range between 2.4 GHz and 10 GHz). The metres were set at Peak and Peak Hold to establish the minimum and maximum levels over the course of 1 h, which were determined to be between 2.2 and 7 mW/m^2^. This is well within the International Commission for Non-Ionising Radiation Protection public guidelines of 10 W/m^2^ for frequencies above 2 GHz within the far field zone averaged over 30 min and the whole body (37).

Monitor and camera units were placed within two metres of the participant’s bedhead depending on their bedroom layout. The baby monitor unit was installed by the investigator within half a metre of the participant’s bedside table and the camera unit was installed at the opposite end of the room, 1.8 to 2 metres of the participant’s bedhead. All baby units appeared identical, whether they were operational or non-operational, as the digital display, microphones and the operating lights were disconnected from both the active and deactivated units. In addition, only the deactivated baby monitor and camera units had their wireless module removed. Participants were randomly assigned to exposure (computer-generated) and fully counterbalanced, with each exposure period separated by a one-week washout period. Double blinding was attained by an independent consultant who programmed the baby monitors (activated or deactivated) in order to mask correct identification of the device status by both participants and investigators. Participants were sequentially provided with a monitor which was designated a random code. In the second intervention week, the codes were alternated to either an active or deactivated (sham) monitor to ensure the reverse condition was met.

### Electromagnetic field measurements in the bedroom

Home visits were conducted to obtain written consent, provide information on the study and to assess electromagnetic field levels in the immediate environment of the bedroom and in particular, on the potential participant’s bed (pillow). The latter was to ensure exposures during sleep would not exceed 0.1 μT for ambient Alternating Current (AC) magnetic fields and were equal to or below 0.02 mW/m^2^ radiofrequency fields (27 MHz to 10 GHz). These levels were derived from the Building Biology Evaluation Guidelines for Sleeping Areas (38). AC magnetic fields were measured with the FM10 Fauser (omnidirectional 3-axis digital gauss metre) and radiofrequencies were measured with the Gigahertz HF59B Analyser with UBB27 antenna (frequency range between 27 MHz to 3.3 GHz) and the Gigahertz HFW59D with UBB2410 antenna (frequency range between 2.4 GHz and 10 GHz). Electromagnetic field readings were also taken on the last day of the trial period to confirm Alternating Current (AC) magnetic fields in the bedroom were below 0.1 μT and radiofrequency fields (27 MHz to 10 GHz) were equal to or below 0.02 mW/m^2^.

### Procedure

**Figure 1:**
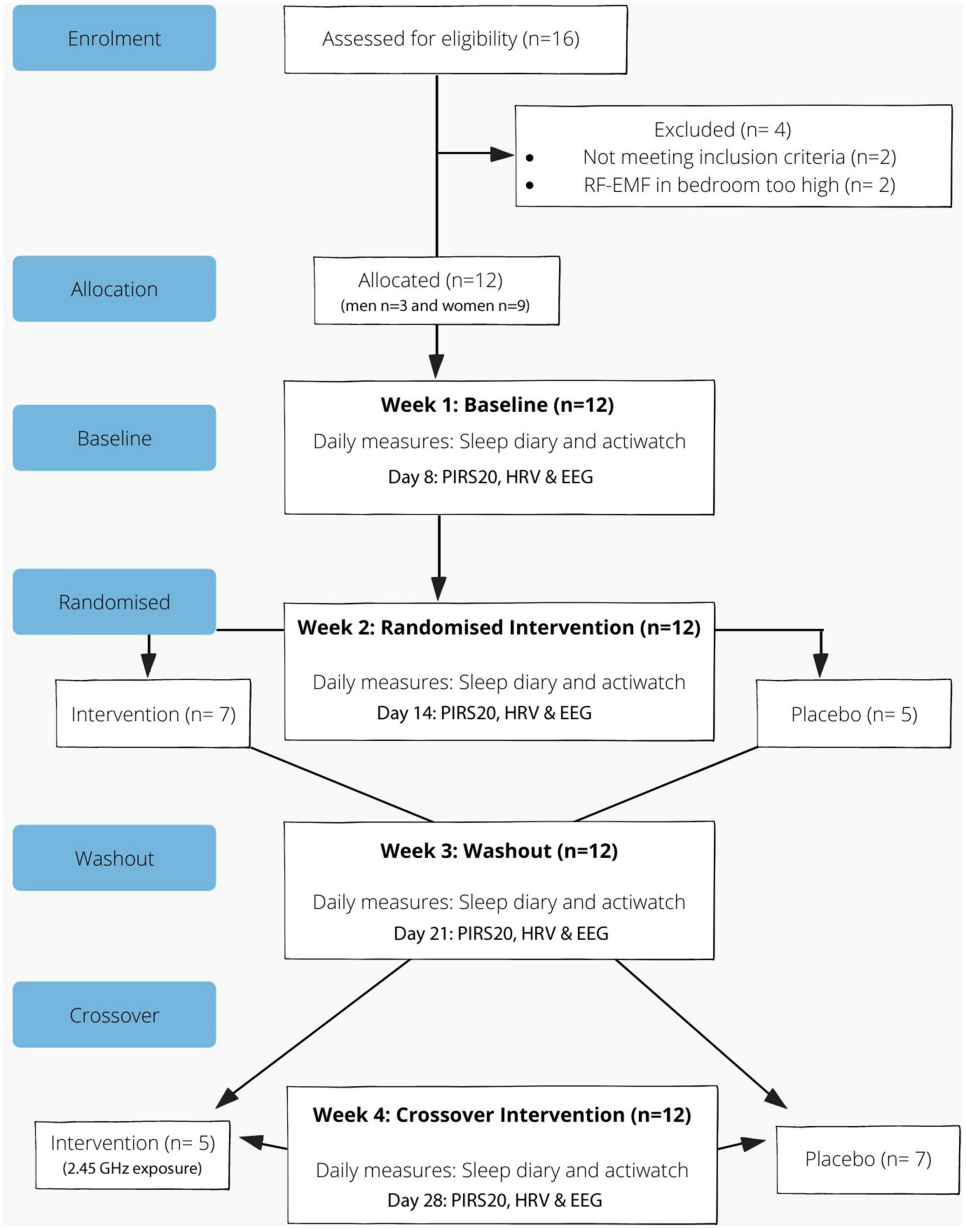
Reporting of trials flow diagram for crossover study involving a baby monitor (intervention).

Participant flow through enrolment, randomisation, follow up and intervention is shown in Figure 1. The 4 week study consisted of baseline (week 1), intervention (weeks 2 and 4) and a washout period (week 3). The procedure included eight home visits with regular communication via text messages throughout the study period to confirm compliance and ensure correct use of the devices. In addition, an instructional booklet and video on the use of the devices was provided to support participants during the study period.

To measure the ambient EMFs in the bedroom, a home visit was conducted in the first week. At this visit, the investigator also explained the data entry process in the daily sleep diary and continuous wearing of the Actiwatch over the course of 4 weeks (except during bathing). A battery charger was provided in weeks 2 or 3 to ensure adequate battery life during the entire study period.

A follow up home visit was conducted at the end of week one to instruct participants on using the PSG (Z-Machine) and heart rate (ECG) monitors. At this visit, the investigator reminded participants to complete the PIRS-20 survey (8th day of the study). Use of the PSG monitor involved cleaning the skin behind the earlobes (mastoid: A1, A2) with an alcoholic swab, and the bony protuberance (spine) at the back of the neck (around C7), attaching the EEG electrodes to these locations, and connecting the wires to the PSG monitor. Participants were shown where to place the device (under the pillow) at night time and how to turn the unit off upon waking. During the same visit, the investigator demonstrated how to use the ECG monitor which involved how to clean and attach the three electrodes on the chest [i.e., right-and left-hand side of the body, collarbone (RA / LA) and rib cage (LL)] and how to attach the leads and turn on the monitor. This practice was replicated at the end of each week (7th night) for the duration of the study. Participants were advised to repeat this procedure at roughly the same time of night for each sleep phase (in total 4 nights across the study period). The monitors were then collected on the 8th day and the investigator downloaded the recorded data for analysis.

To ensure adequate battery life for the week, on the first day of interventions weeks (2 and 4), both the monitor and camera units were connected to a power socket. In situations where the socket was not in close proximity to the bedhead and/or the opposite end of the room, an extension lead was used. The monitors were collected on day 8.

A final home visit was conducted at the end of the last week of the study (day 28), to take a final measurement of the ambient EMFs in the bedroom. Blinding was assessed by asking participants which week they thought they received the active intervention (week 2 or 4).

### Participants

A power calculation was conducted to estimate the preferred sample size for the study. The calculation of effect size estimates were derived from the Lustenberger et al. (24) study where the exposure to RF-EMF pulses resulted in a significant reduction in sleep time (Mean decrease 9.23 min, SD 13.6). Therefore, based on this result, to achieve at least 80% power (G*Power 3.1.9.2) at alpha level of 0.05, a minimum sample of 20 participants is required (39).

Inclusion criteria were based on age (18 to 56 years), location (lived in a detached home in Melbourne), the absence of existing sleep disturbances or conditions that may affect sleep (pre-existing illness, bed partner, light, noise), being a non-smoker, speaking English and ability to provide informed consent. Participants that were taking any medications or supplements or on antibiotic therapy, diagnosed with any chronic condition, recently hospitalised or had surgery, wore a pacemaker, worked nightshifts, had to travel across time zones 2 weeks before or during the study period, had to use a mobile phone during the night, pregnant or peri or post-menopausal, unable to provide informed consent, smoked or had a BMI over 30 or any other condition that impacted sleep were excluded. In addition, participants were excluded if their bed was adjacent to a smart metre, metre panel or inverter, and/or if they had Wi-Fi enabled devices, cordless phones, extenders, or boosters in their bedroom that they were not willing to relocate. Participants were also excluded if the ambient EMF measurement in their bedroom before and after the study, exceeded 0.1 μT or 0.02 mW/m^2^. Participants were advised to avoid using digital devices for at least an hour prior to bedtime, go to bed and wake up at about the same time over the study period and abstain from drinking alcohol or caffeine in the afternoon (after 3 pm).

Participants were recruited via an advertisement campaign on social media. Eligibility for the study was assessed using the Participant Eligibility Screening Questionnaire according to the exclusion and inclusion criteria. Participants deemed eligible were provided with the Participant Information and Consent Form. This was followed with a phone call to address questions regarding participation in the study and organise a convenient time to visit the home.

Between October 2019 and March 2020, 12 adults consisting of 3 men and 9 women participated in the study. The mean age of the females was 41 (SD ±9) and males was 47 (SD ±3) and the mean BMI for females was 22.9 kg/m^2^ and males was 24.6 kg/m^2^.

### Measures

#### Pittsburgh Insomnia Rating Scale (PIRS-20)

The Pittsburgh Insomnia Rating Scale survey was used to assess subjective sleep quality on the 8th day of each study-week (four times in total over the study period). PIRS-20 provides an index of insomnia severity with a change in score > 20 considered to be clinically significant (40).

#### Actigraphy

Objective sleep measures were obtained using portable polysomnography and wrist actigraphy combined with a sleep diary. Actigraphy data was collected using a battery-operated wrist actigraphy watch (wGT3X+, Actigraph Pty Ltd) with a solid state piezo-electric accelerometer to generate movement-based voltage and activity counts per epoch. Participants were instructed to wear the Actiwatch for 24 h a day on their non-dominant hand, and data were collected at 30-s epochs. Consistent with recommended standard research guidelines (41), the following objective sleep measures were obtained from this device: sleep onset latency (SOL), sleep efficiency (SE), total sleep time (TST) and wake time after sleep onset (WASO). Actigraphy scoring was done using Cole Kripke algorithm and manually checked against a sleep diary created by the investigator to document when they turned the lights off and went to sleep, the time they woke up, the time they woke up during the night and reasons for this (noise, light, illness, bed partner, kids, temperature etc.), and the amount of time they spent on a digital device (screen time) for the day.

#### Polysomnography

Sleep efficiency, sleep latency, sleep time, sleep staging and EEG power spectrum were measured using a portable single-channel polysomnographic monitor (Zmachine^®^ Insight Model: DT-200, General Sleep Corporation) which collects high quality, objective, epoch-by-epoch, sleep state information and summary sleep statistics (42). The EEG signal for each 30-s epoch is categorised into five categories within the Z-machine algorithm: (1) Wake; (2) Light sleep (N1 & N2 Stages); (3) Deep sleep (N3 Stage); (4) Rapid eye movement sleep (REM-sleep); and (5) sensor connection failures. The sensitivity of the Z-machine algorithm is 95.5% and the specificity is 92.5% compared to other polysomnographic technology (Kaplan et al., 2014; Wang et al., 2015). Following standard procedures with EEG recordings, we engaged in methods to reduce the impact of EMF interference. The device includes patient grounding and 50 Hz notch filter to reduce ambient RF interference with the EEG signal. Also, all raw EEG signals were recorded with <5 KOhm impedence and visually inspected for anomalies by an PSG technician with over 20 years experience.

#### Heart rate variability

Heart Rate Variability (HRV) was used to measure the autonomic nervous system (ANS) through a battery-operated portable ECG monitor (Contec TLC9803) that had no Bluetooth or Wi-Fi capability. Analysis of HRV was performed in 5-min samples at baseline, washout, and intervention weeks at similar time of night for each sleep phase. The HRV domains of time and frequency were analysed using Kubios (v 3.0.1, Biosignal Analysis and Medical Imaging Group, Finland). The HRV index was derived from the Root Mean Square of Successive Differences in R-R intervals (RMSSD). In order to quantify the sympathovagal balance levels between sympathetic and parasympathetic activity, the mean ratio of low frequency (0.04–0.15 Hz) to high frequency (0.15–0.4 Hz) HRV power (LF/HF) was used. An index of ANS reactivity to intervention was calculated using the following equation: (increase of HRV or LF/HF ratio from baseline to intervention/baseline HRV or LF/HF ratio)*100.

### Statistical analysis

All analyses were performed using the Statistical Package for the Social Sciences software (SPSS Inc., Version 28, Armonk, New York, United States). Differences between the Intervention and Placebo EMF Exposure were analysed using paired samples t-test with a *p*-value less than 0.05 considered as statistically significant.

## Results

**Figure 2:**
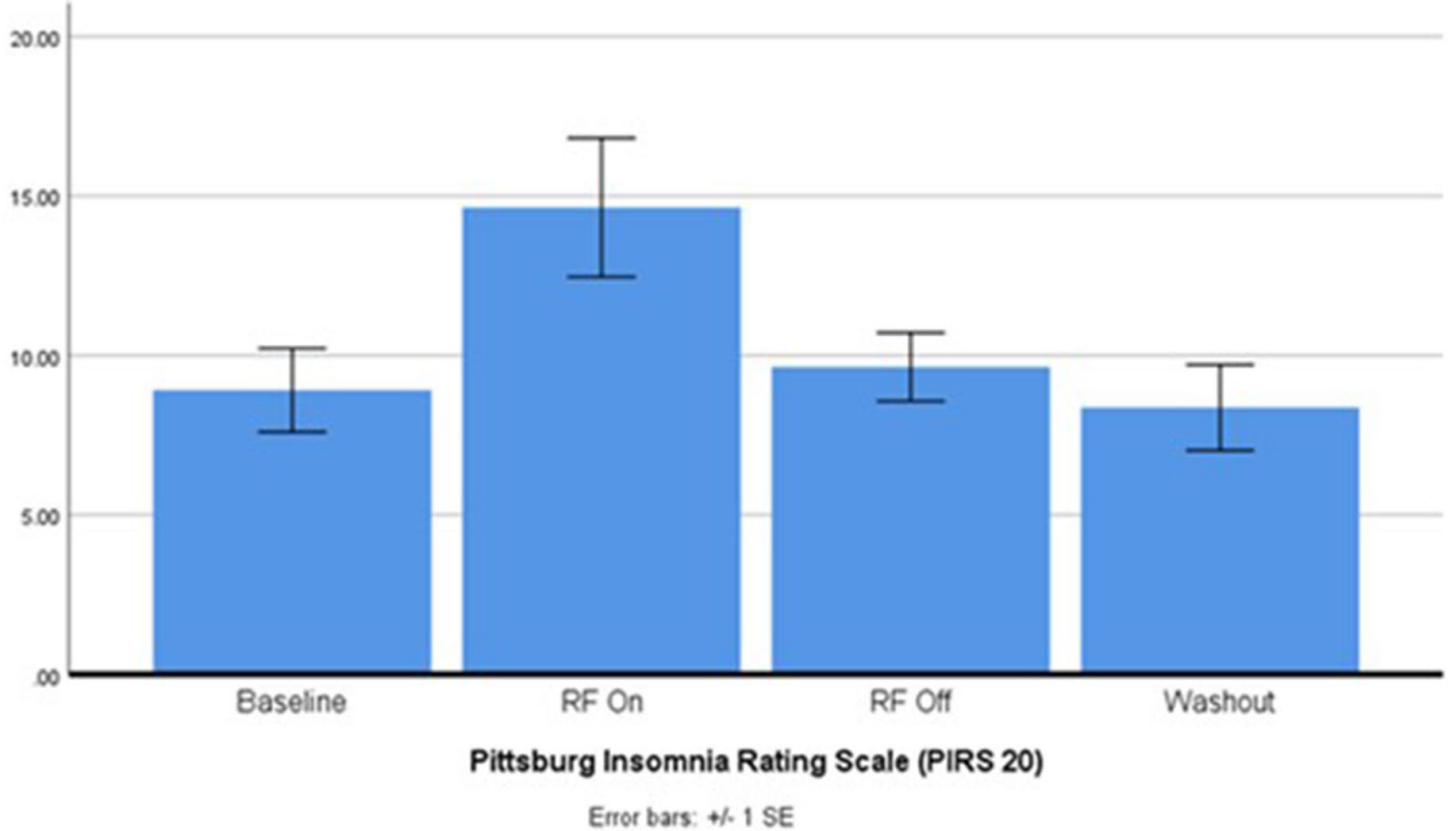
Pittsburgh insomnia rating scale after 2.45 GHz RF-EMF exposure (*p* < 0.004). ANOVA: E(3, 30) = 5.457, *p* = 0.004, partial n2 = 0.35. *Post-hoc* pairwise EMF on vs. EMF off *p* = 0.03.

While the goal was to recruit 20 participants, due to the strict inclusion criteria and impact of the pandemic, data from only 12 participants were evaluated. Summary statistics for the primary and secondary outcome measures are outlined in Table 1. Sleep quality as indicated by the PIRS-20 was found to be significantly reduced during RF-EMF exposure compared to placebo exposure (*p* < 0.05) as illustrated in Figure 2. Three participants (27.3%) scored above the cut off of 20 (out of a total score of 60 for PIRS-20) for risk of clinical insomnia. The raw single-channel EEG signal derived from the Z-machine was converted to EDF format and analysed using Curry 7 EEG analysis software (Compumedics Pty Ltd). The EEG signal was high/low pass filtered (.3 Hz/70 Hz) with a 50 Hz notch filter. Compared to sham-exposure, during the RF-EMF exposure, a statistically significant increase (*p* < 0.5) in EEG power density in the higher frequencies (theta, beta and gamma bands) was detected during Non-Rapid Eye Movement (NREM) sleep but not in Rapid Eye Movement (REM) sleep. No differences were observed in HRV or actigraphy. When asked, only 44% correctly identified the week with the active intervention.

**Table 1:** Summary statistics for primary and secondary sleep outcome measures.

	Baseline Week 1 *Mean ± SD*	Washout Week 3 *Mean ± SD*	Intervention on *Mean ± SD*	Intervention off *Mean ± SD*	*n*	*t*-statistic, *p*-value; Bootstrap *95% CI*	Effect size (Cohen’s *d*)
Primary outcome							
PIRS-20	8.91 ± 4.35	8.36 ± 4.46	14.64 ± 7.21	9.63 ± 3.56	11	*t* = 2.48, *p* = 0.03*; BCa [1.18, 8.73]	*d* = 0.75
Secondary outcomes							
Number of awakenings (NOA)	17.73 ± 3.94	15.60 ± 6.32	16.37 ± 3.00	16.10 ± 6.89	11	*t* = 0.11, *p* = 0.46; BCa [−2.96, 4.14]	d = 0.04
Actigraphy							
TST	409.37 ± 42.55	397.35 ± 56.78	412.99 ± 27.78	406.38 ± 74.85	8	*t* = 0.29, *p* = 0.78; BCa [−63.92, 53.06]	*d* = 0.10
SE	89.38 ± 4.01	90.25 ± 3.23	89.57 ± 3.41	89.66 ± 6.85	8	*t* = −0.32, *p* = 0.97; BCa [−5.97, 5.27]	*d* = 0.02
WASO	48.56 ± 21.24	40.83 ± 14.30	46.43 ± 15.26	38.80 ± 17.91	8	*t* = 0.93, *p* = 0.47; BCa [−2.90, 19.27]	*d* = 0.33
Polysomnography							
SOL	26.60 ± 17.28	15.33 ± 9.16	22.74 ± 14.79	22.92 ± 14.99	10	*t* = −0.05, *p* = 0.96; BCa [−6.00, 6.06]	*d* = 0.02
TST	405.94 ± 55.91	405.47 ± 56.74	396.33 ± 43.02	378.42 ± 68.75	10	*t* = 0.72, *p* = 0.49; BCa [−32.28, 60.96]	*d* = 0.23
SE	83.61 ± 4.54	86.14 ± 5.53	82.77 ± 8.78	84.15 ± 4.83	10	*t* = −0.60, *p* = 0.56; BCa [−6.00, 3.03]	*d* = 0.19
WASO	44.46 ± 27.50	39.26 ± 34.66	50.64 ± 46.47	40.65 ± 28.25	10	*t* = 0.94, *p* = 0.38; BCa [−5.46, 27.51]	*d* = 0.30
SWS time	62.06 ± 38.23	67.00 ± 37.21	80.43 ± 23.74	66.12 ± 27.32	10	*t* = 1.67, *p* = 0.13; BCa [−1.41, 32.16]	*d* = 0.53
REM time	99.07 ± 35.00	110.47 ± 40.17	110.52 ± 33.34	99.48 ± 30.61	10	*t* = 0.81, *p* = 0.44; BCa [−14.94, 39.72]	*d* = 0.26
Heart rate variability							
SWS RMSSD	47.28 ± 19.09	42.50 ± 19.84	39.23 ± 20.10	29.00 ± 15.30	8	*t* = 1.73, *p* = 0.13; BCa [−3.31, 18.13]	*d* = 0.61
SWS LF/HF ratio	2.19 ± 2.78	1.17 ± 0.63	1.24 ± 1.05	3.62 ± 8.19	8	*t* = −0.84, *p* = 0.43; BCa [−8.19, 0.67]	*d* = 0.30
NREM RMSSD	31.70 ± 4.98	40.07 ± 16.24	67.34 ± 65.93	36.68 ± 23.78	8	*t* = 1.27, *p* = 0.24; BCa [−0.15, 70.25]	*d* = 0.45
NREM LF/HF ratio	2.88 ± 3.56	3.46 ± 3.90	4.53 ± 6.49	1.58 ± 1.24	8	*t* = 1.21, *p* = 0.26; BCa [−1.00, 7.72]	*d* = 0.50
REM RMSSD	41.47 ± 13.34	36.27 ± 12.89	49.74 ± 16.86	41.76 ± 32.67	8	*t* = 0.99, *p* = 0.36; BCa [−10.00, 23.27]	*d* = 0.35
REM LF/HF ratio	1.75 ± 0.89	1.87 ± 1.92	2.11 ± 1.33	1.33 ± 0.65	8	*t* = 1.42, *p* = 0.20; BCa [−1.00, 1.652.11]	*d* = 0.55
Electronic device use (Hours/Week)	24.07 ± 14.89	24.60 ± 15.25	20.37 ± 8.85	21.45 ± 9.55	9	*t* = 0.46, *p* = 0.66; BCa [−5.85, 3.57]	*d* = 0.15
NREM EEG power density (μV^2^)							
Delta (1-3 Hz) EEG power density	0.48 ± 0.37	0.33 ± 0.31	0.53 ± 0.45	0.55 ± 0.42	10	*t* = 0.19, *p* = 0.92; BCa [−0.32, 0.39]	*d* = 0.03
Theta (3-8 Hz) EEG power density	0.05 ± 0.08	0.07 ± 0.19	0.36 ± 0.36	0.08 ± 0.15	10	*t* = −2.76, *p* = 0.04*; BCa [−0.48, −0.11]	*d* = 0.87
Alpha (8-13 Hz) EEG power density	0.04 ± 0.05	0.07 ± 0.17	0.32 ± 0.35	0.11 ± 0.22	10	*t* = −1.97, *p* = 0.16; BCa [−0.44, −0.04]	*d* = 0.63
Beta (13-30 Hz) EEG power density	0.05 ± 0.04	0.08 ± 0.19	0.67 ± 0.69	0.07 ± 0.09	10	*t* = −2.95, *p* = 0.03*; BCa [−1.05, −0.22]	*d* = 0.93
Gamma (30-70 Hz) EEG power density	0.08 ± 0.07	0.21 ± 0.53	1.06 ± 1.04	0.24 ± 0.40	10	*t* = −3.24, *p* = 0.02*; BCa [−1.29, −0.37]	*d* = 1.04

Results from the primary outcome measure of this study (Pittsburgh Insomnia Rating Scale-20), are shown in this table. Secondary outcome measures included Actigraphy, Polysomnography, Heart Rate Variability (HRV), and EEG Frequency Analyses from NREM sleep. Heart Rate Variability and EEG analyses were derived from 5-min samples matched for time of night (within 60 min) within sleep stages across conditions. KEY: PIRS-20-Pittsburgh Insomnia Rating Scale-20; TST-Total Sleep Time; SE-Sleep Efficiency; WASO-Wake After Sleep Onset; SOL-Sleep Onset Latency, SWS-Slow Wave Sleep; REM-Rapid Eye Movement; NREM-Non-Rapid Eye Movement; EEG-Electroencephalogram. *Indicates *p* < 0.05.

There were a few instances where the equipment was not activated correctly and/or non-compliance was an issue. Actigraphy was not collected for four participants across the study period due to equipment failure and/or non-compliance during some of the study period (*n* = 8). PSG was not collected for two participants due to equipment failure (*n* = 10). One participant came down with a flu-like illness in week 4 (intervention OFF) and their PIRS-20 data was not included for that week (*n* = 11).

## Discussion

This study is the first double-blind, randomised, placebo-controlled study to report the impact of exposure to a multi-night radiofrequency device (baby monitor) on clinically relevant sleep outcomes under real-world conditions. The results of the PIRS-20 reveal that 7 consecutive all-night exposure to RF-EMF led to reduced subjective sleep outcomes with three participants (27.3%) scoring above the threshold for risk of clinical insomnia. Poorer subjective sleep outcomes as measured by the Karolinska Sleepiness Scale has been reported following a 3-h exposure to a mobile phone 884 MHz (43). In contrast, studies involving near field exposures to a 900 MHz frequency over six nights using Pittsburgh Sleep Quality Index (22) or operator-recorded mobile phone use (GSM/UMTS network) at baseline, and sleep outcomes at baseline and at the 4-year follow-up using the Medical Outcome Sleep Questionnaire (27), did not report significant effects on sleep.

Despite the small sample size and the study being potentially underpowered for detecting differences in objective measures, during Non-Rapid Eye Movement (NREM) the statistically significant changes in the PIRS-20 coincided with a statistically significant increase in theta, beta, and gamma EEG power density between conditions. These findings suggest there are large effect sizes relative to the noise in these measures and are consistent with research on mobile phone exposure, which shows significant modification of the alpha band (44) and increased power of various frequencies (23, 26, 28, 43, 45). A recent systematic review reported that the EEG power in the alpha frequency range increased in 10, diminished in four, and not altered in eight studies (46). Another review concluded that the mechanism by which RF-EMFs may impact sleep is likely to be due to an increase in the electroencephalogram power when exposure occurs immediately prior to or during sleep (20). While the EEG power in the alpha frequency range was not statistically significant in this study, the effect size of *d* = 0.63 (Table 1), 95% power with alpha of 0.05 level, suggests a projected sample of 35 using G*Power would be required to detect a significant difference between the exposure and sham exposure conditions. It has been suggested that the impact of RF-EMF exposure on sleep related outcomes are more likely to be observed during extended time (>30 min) and the entire night time (21). While this is consistent with our findings, nonetheless it is difficult to draw definitive conclusions as there are many complicating and confounding factors. Furthermore, despite initial efforts to maintain a balanced design, various factors including participant attrition, incomplete data sets, and technical issues led to unequal group sizes across different order conditions.

The statistically significant mean differences observed in the PIRS-20 was largely affected by three participants reporting clinical levels of insomnia risk during the RF-EMF exposure condition. All three were women in their 40s and 50s. This observation aligns with previous research indicating that older women exhibit a higher likelihood of electrical sensitivity (13, 47). While gene variants reported to be associated with EMF sensitivity do not appear to be gender-specific, they are related to DNA repair mechanisms, oxidative stress (GSTT1 and GSTM1 variants), and detoxification and drug metabolism pathways (CYP2C19*1/2) (48). These genetic factors may explain the frequent co-occurrence of Multiple Chemical Sensitivity with Electromagnetic Hypersensitivity (EHS) (13). While we did not undertake genetic testing in the participants, the role of age in EMF sensitivity may be attributed to the reduced ability of older adults to repair cellular damage resulting from long-term exposure to environmental stressors. However, this factor alone does not account for the unique susceptibility observed in some women and none of the participants in this study had Multiple Chemical Sensitivity or were involved in occupations that involved long term or high exposures to electromagnetic fields. These findings underscore the complexity of EMF susceptibility and highlight the need for further investigation into individual differences. Future research should consider participants’ genetic variants, exposure history (occupational exposure, medical history, e.g., X-rays and MRIs); place history (proximity to known external sources, e.g., mobile phone base stations and high-voltage transmission lines) and personal EMF exposure monitoring. Such comprehensive approaches would contribute to a more nuanced understanding of the factors influencing EMF sensitivity and its associated health effects.

Comparing our results to the findings of previous studies is a significant challenge because most studies on RF-EMF and sleep have focused on short-term exposure to mobile phone frequencies under simulated conditions in laboratory settings, or epidemiological surveys prone to respondent bias (20, 49). Two reviews conducted a decade ago, concluded that there is lack of evidence for a direct link between mobile phone exposure and severity of non-specific physical symptoms such as sleep problems (50, 51). However, this contradicts a growing number of systematic reviews that have reported pulse-modulated RF-EMFs related to altered brain physiology indicated by changes in electroencephalogram power in selective bands (alpha, beta, delta or theta) when administered immediately prior to or during sleep (20, 31, 33, 52). The heterogeneity between studies appears to be due to multiple factors including differences in study design, timing of exposure relative to sleep, as well as proximity and duration of exposures. In addition, the type of radiofrequency devices employed, the type of frequency used, modulation, power density, field strength, pulsing nature, challenges in controlling extraneous confounding factors, varying criteria for participant inclusion, statistical power and bias, and the laboratory or clinical context involved also vary widely between studies.

The impact of commonly used Blue-Tooth and Wi-Fi compatible devices such as routers, baby monitors and smart phones on clinically relevant sleep indicators has not been widely studied. To date only two studies examining the effects of Wi-Fi frequency exposure (using 2.45 GHz frequency band) on sleep have been published with mixed results and these have been performed in simulated laboratory settings rather than in a real-world context. A study involving a one off 60-min Wi-Fi exposure in healthy adults resulted in no changes to the spectral power of spontaneous awake electroencephalographic activity (53), while another study reported that a single night exposure to a Wi-Fi router in a sleep laboratory resulted in a reduction in the alpha frequency band of the global EEG power during NREM with no change in subjective sleep parameters (54). In the present study, a statistically significant increase in theta, beta, and gamma EEG power density during NREM sleep was observed alongside a significant reduction in subjective sleep quality with multi-night exposure to 2.45 GHz radiation. Although speculative, it is possible that this observed change in NREM EEG is related to poorer subjective sleep quality due to increased cortical arousal in NREM sleep (55) or other mechanisms that are currently unknown.

### Strengths and limitations

The study had several strengths including the robust randomised, double-blind, placebo-controlled, crossover design and the inclusion of healthy adults in a real-world context. Application of a commercially available RF device designed to be placed in the bedroom over seven consecutive all-nights and the use of a clinically relevant measure of sleep as the primary outcome also provides ecological validity. While variability between placements of the camera and monitor units is likely to impact exposure received by the participants, each participant acted as their own control across the two conditions, and spot measurements conducted on the participant’s bed at the beginning and end of the study confirmed exposures did not exceed 0.1 μT and 0.02 mW/m^2^.

There are limitations of this study that arise from the real-world conditions, including the inability to control extraneous variables such as the participant’s behaviour and the need to account for exposures to multiple devices during the day which could have confounding effects. In addition, even though exposure levels in the bedroom of each participant was assessed before and after the study, continuous monitoring of RF-EMF exposure was not undertaken. The multiplicity of analyses may indicate the finding of a reduction of PIRS-20 with NIR-EMF exposure could be due to chance. It also highlighted, despite only recruiting 12 participants, the effect size for the PIRS-20 could be considered large (*d* = 0.75), whereas the effect size observed for a range of objective measures varied between 0.02 and 0.61 (small and medium).

Extrapolating the results of this study to exposure from devices that employ different frequencies and/or modulations is a challenge. It has been suggested that modulated or pulsed RF-EMFs are more bioactive than non-modulated or non-pulsing fields of the same carrier frequency and of the same average intensity (49). The devices used in our study used an operating frequency range between 2.400 ∼ 2.4835 GHz similar to many Wi-Fi enabled devices, however the modulation used was Gaussian Frequency Shift Keying (GFSK) with a frequency-hopping spread spectrum (FHSS). Given these features, our findings may be more relevant to devices that employ GFSK modulation such as GSM, DECT and Personal Area Networks such as Bluetooth and wearables (56).

Another limitation that arose because the study was conducted at home, was that the EEG recording was limited to a single channel portable EEG system, which does not provide the same precision in calculating global EEG spectral power as multi-electrode lab-based studies. Furthermore, the small sample size (*n* = 8–12) means that the study was underpowered to detect small differences in subjective and objective measures. The finding of statistically significant effects for the PIRS-20 (*d* = 0.75) and increased electroencephalogram (EEG) power suggest large effect sizes. Since our sample consisted of only healthy adults, caution should be exercised in generalisability to other age groups or clinical populations. A larger follow-on study would need to consider limiting the number of secondary measures to reduce inflation of type 1 error rate due to multiple comparisons. For example, actigraphy did not appear to provide the accuracy or fidelity of sleep assessment required (as it is based on movement algorithms), so this measure is not recommended in follow-up studies.

## Conclusion

Our preliminary findings demonstrate that radiofrequency devices induce statistically significant changes in the EEG during Non-Rapid Eye Movement (NREM) sleep and suggest these devices may have a clinically important adverse effect on sleep in some people in real-world scenarios. In light of the small sample size and limitations of the study, further large-scale investigations are required to confirm these findings. Future studies that account for individual variances such as gender, age, genetic variants, occupational, medical and exposure history, would help identify at risk individuals. Furthermore studies that include exposure dosimetry, placement of exposure devices that are well-defined, consistent, and consider signal features such as modulation, field strength, resonance, pulsing, polarisation and power flux density would provide more detail regarding the types of devices that may produce adverse effects under real world scenarios. Until further studies verify or provide evidence contrary to these findings, caution is advised when using RF-EMF devices in bedrooms.

## Data Availability

The raw data supporting the conclusions of this article will be made available by the authors, without undue reservation.
